# Mental and behavioral health care access and satisfaction among older sexual minority women

**DOI:** 10.20935/mhealthwellb7867

**Published:** 2025-08-26

**Authors:** Jillian R. Scheer, Gabriella Epshteyn, Rachel Girard, Michelle A. Stage, Elizabeth M. Chadbourne, Billy A. Caceres, Melanie M. Wall, Tonda L. Hughes

**Affiliations:** 1 Department of Psychology, College of Health Sciences, University of Rhode Island, Kingston, RI, USA.; 2 Center for Sexual and Gender Minority Health Research, School of Nursing, Columbia University, New York, NY, USA.; 3 Columbia University School of Nursing, Columbia University, New York, NY, USA.; 4 Department of Psychiatry, Vagelos College of Physicians and Surgeons, Columbia University Irving Medical Center, New York, NY, USA.; 5 Department of Biostatistics, Mailman School of Public Health, Columbia University Irving Medical Center, New York, NY, USA.

**Keywords:** sexual minority women, older women, help-seeking, treatment satisfaction, mental health, behavioral health

## Abstract

This study expands upon syndemics, structural vulnerability, and health care utilization frameworks by examining individual and social factors influencing help-seeking, care barriers, and treatment satisfaction among sexual minority women (SMW; e.g., bisexual, lesbian) aged 50+ from Wave 3 of a community-based longitudinal study, the Chicago Health and Life Experiences of Women Study. Wave 3 data were collected in 2010–2012 (May 2010 to August 2012). Bivariate analyses were conducted to examine associations among help-seeking behaviors, self-rated physical and mental health, relationship status, and social support. We used multivariate logistic regression models to examine (1) associations between sociodemographic characteristics and help-seeking, (2) associations between help-seeking and treatment satisfaction variables and sociodemographic characteristics, (3) self-rated physical health and self-rated mental health as predictors of help-seeking and treatment satisfaction, and (4) interpersonal factors as predictors of help-seeking and treatment satisfaction. Participants (*N* = 196) were, on average, 58.15 years of age (SD = 6.61). Physical health and issues related to depression, anxiety, memory, and eating are synergistic among older SMW. Those with insufficient household incomes and identifying as bisexual, Black, or younger were more likely to seek help. Black and Latinx SMW reported fewer help-seeking barriers than White SMW. Self-rated poorer mental health was associated with greater difficulty accessing services; sociodemographic variables weakened this association. Poorer mental health and lower social support were associated with treatment dissatisfaction. Results highlight the relevance of structural vulnerabilities and social determinants of health among older SMW, emphasizing the need for integrated, culturally competent care models that enhance access and improve treatment satisfaction.

## 1. Introduction

Older sexual minority women (SMW; e.g., bisexual, lesbian) remain a largely invisible and understudied segment of the older adult population, even as the number of SMW is growing rapidly [1, 2]. Despite the paucity of research on older SMW’s health, existing studies demonstrate significant health disparities (e.g., mood and anxiety disorders, cognitive impairment, unhealthy substance use) linked to sexual identity [2–4]. This elevated risk stems from compounding stressors that disproportionately impact older SMW, for example, aging- and identity-based stigma, socioeconomic disadvantages, and violence [5, 6]. Such stressors contribute to increased health needs [7]; likely a reason that aging SMW access treatment, such as counseling, more frequently than their heterosexual peers [8]. Older SMW face unique barriers to accessing care and report treatment dissatisfaction, such as greater difficulty accessing appropriate care and a lower satisfaction with the services they do receive [9]. Yet, few studies have examined help-seeking, care barriers, and treatment satisfaction among older SMW, hindering efforts to improve treatment access and effectiveness for this population.

### 1.1. Help-seeking behavior and barriers to accessing needed care among older sexual minority women

Mood and anxiety disorders, cognitive decline, and unhealthy substance use are *syndemic* among older SMW, co-occurring and compounding negative health outcomes beyond any single issue [10]. Shaped and exacerbated by harmful social conditions, such as poverty, these issues significantly increase disease burden in this population [11]. Studies of older populations in the United States using nationally representative and state-wide samples found that depressive symptoms were associated with higher odds of cognitive impairment among sexual minority people, but not among heterosexual people [11], and that SMW were more likely than heterosexual women to report multiple health conditions, including depression and eating disorders [12]. Yet, research on help-seeking among older SMW has focused on service use in general, visit frequency, or help-seeking for single conditions [13]. This narrow focus fails to capture the complex reality of synergistic health issues faced by older SMW [14].

Compounding the likelihood of multiple health concerns, older SMW are more likely than older heterosexual women to report barriers to accessing mental and behavioral health care [13]. For example, compared to their heterosexual counterparts, older SMW report more structural obstacles, such as limited geographic and financial accessibility and lack of continuity in care [13]. Older SMW are also more likely to encounter providers with heteronormative assumptions, biases, and limited understanding of their unique needs, further deterring them from accessing needed care [15]. Such delays may lead older SMW to seek help only when symptoms become severe, increasing their vulnerability to chronic health issues [3, 15]. Despite higher treatment-seeking rates, older SMW report more unmet health needs than their heterosexual counterparts [15]. These unmet needs contribute to reduced quality of life, increased morbidity, and years of potential life lost [15].

### 1.2. Structural vulnerability and help-seeking frameworks

**Figure 1 •:**
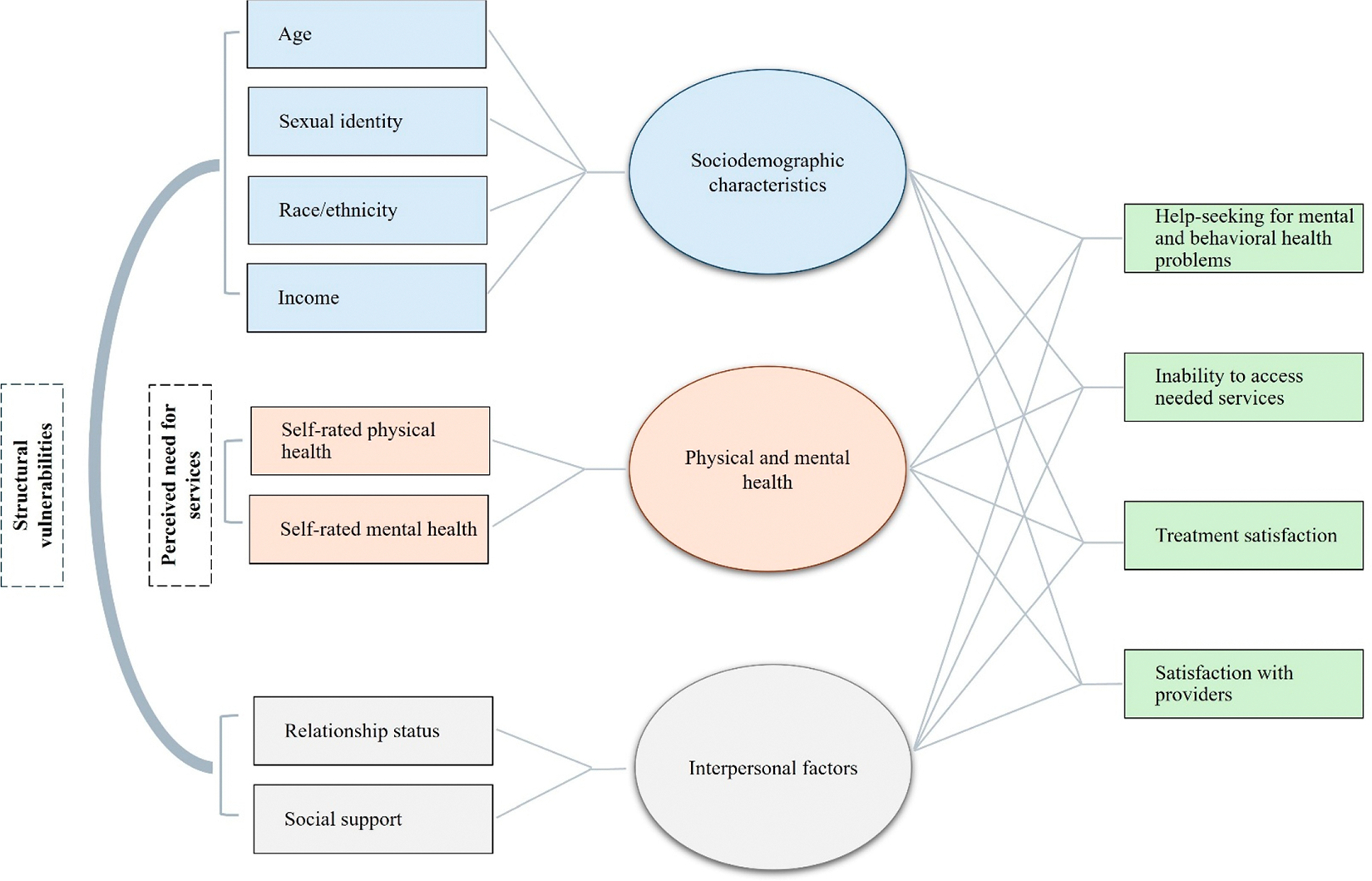
Conceptual model of structural vulnerabilities, perceived need for services, help-seeking, and treatment satisfaction among older sexual minority women. Note: This conceptual model illustrates the multifaceted factors influencing mental and behavioral health service outcomes among older sexual minority women (SMW). It adapts the behavioral model of healthcare utilization to highlight the role of structural vulnerabilities and perceived need for services as overarching contextual influences. These factors are reflected in three main domains: sociodemographic characteristics (e.g., age, sexual identity, race/ethnicity, income), physical and mental health (e.g., self-rated health), and interpersonal factors (e.g., relationship status, social support). Arrows from each domain point to four key health service outcomes—help-seeking, inability to access needed services, treatment satisfaction, and satisfaction with providers—indicating that these outcomes are shaped by the interplay of individual, interpersonal, and systemic factors. The model underscores the importance of addressing both social determinants and psychological needs to improve health service engagement and satisfaction among older SMW.

Help-seeking behaviors and barriers to accessing services likely vary among older SMW. Informed by public health theories, the concept of *structural vulnerability* [16, 17] describes how social, economic, and cultural factors—such as sociodemographic characteristics and social networks—influence help-seeking behavior and health care access. Indeed, sexual identity-related health disparities and healthcare access inequities among aging populations are often driven by inadequate or lack of insurance, insufficient financial resources, reduced social support, and structural inequities tied to ageism, heterosexism, and racism [13]. Despite elevated risks faced by older SMW [3, 9, 13], few studies have explicitly used the structural vulnerability framework in research with this population (see Figure 1). The existing literature on sexual and gender minority older adults, including SMW, has explored how key life events, relationship status, and social networks relate to health, well-being, and quality of life, while also examining disparities linked to race/ethnicity, depression, and high-risk drinking. Studies have also identified psychological, behavioral, and social mechanisms that contribute to health equity and healthcare engagement in this population [3, 9, 13]. The structural vulnerability framework may be useful in improving our understanding of whether and how help-seeking behaviors and barriers to accessing services vary by sociodemographic characteristics and interpersonal factors among older SMW.

Importantly, older SMW who experience multiple forms of marginalization—such as being racial or ethnic minorities or are of low-income—may face more pronounced barriers to accessing health services, including provider bias, transportation challenges, and systemic exclusion from affirming care settings [9]. Yet, it is also possible that some older SMW with multiple minoritized identities have cultivated strong informal support systems or have accessed services through identity-specific networks that may reduce perceived barriers to care. Thus, while structural vulnerability can compound disadvantages, it can also intersect with community strengths and protective factors, shaping diverse patterns of help-seeking and engagement in care among older SMW [18].

One aspect missing from the concept of structural vulnerability is the perceived need for services. According to Andersen’s behavioral health care utilization model [19, 20], perceived need for services based on functional health and symptom severity predicts help-seeking for mental and behavioral health issues [19, 20]. Studies have found that older adults with physical and mental health concerns were more likely to seek help than those without such conditions [20]. Among SMW in general, health concerns are positively associated with service use [21]. At the same time, women who report more mentally and physically unhealthy days and chronic diseases also report delaying care and experiencing barriers to accessing needed services compared to their counterparts [22]. Therefore, older SMW with more pressing health needs likely have both greater perceived need for services and greater barriers to accessing needed services.

### 1.3. Treatment satisfaction among older sexual minority women

Treatment satisfaction is another critical yet understudied dimension of care for older SMW and serves as a key metric for healthcare quality [23]. Higher treatment satisfaction is associated with better compliance with treatment recommendations, improved well-being, reduced dropout rates, and better treatment outcomes [24]. Prior work has shown that satisfaction with treatment and healthcare providers is influenced by patients’ trust, comfort in communicating concerns, and involvement in treatment decisions [25].

Variations in treatment satisfaction among aging populations can be explained in part by patient characteristics, such as sociodemographic factors, and presenting concerns [26]. For instance, a study of over 1000 patients with type II diabetes found that those who reported fewer complications experienced higher treatment satisfaction [24]. Notably, women had lower scores than men on perceived well-being and treatment satisfaction [24]. Among older adults in general, research indicates lower levels of satisfaction with healthcare services and provider interactions, particularly among those with less adequate health literacy [26].

Factors driving differences in treatment satisfaction among older SMW are poorly understood. Research from population-based surveys and convenience samples of treatment-seeking populations consistently shows that SMW report lower treatment satisfaction compared to their heterosexual counterparts [27]. This disparity is largely driven by unmet health needs, discontinuity in care, poor communication with providers, reluctance to disclose sexual identity, and providers’ insufficient cultural competence in caring for sexual minority people [9, 28]. Structural barriers further compound these challenges. In a study of providers of care for older adults and administrators, nearly 90% were unaware of having served sexual or gender minority clients [29]. In the same study, aged care providers and administrators reported that there are few services specific to the needs of older sexual and gender minority adults [29].

Treatment satisfaction disparities are particularly pronounced for some subgroups within the SMW population. For example, bisexual women report lower satisfaction with healthcare compared to monosexual SMW, potentially due to experiences of biphobia, identity erasure, and invalidation from providers, in addition to experiences of heterosexism, that monosexual SMW may face. Bisexual women may feel that their identities are misunderstood, dismissed, or pathologized, which can undermine trust in providers and the perceived quality of care. Similarly, SMW of color often face intersecting forms of discrimination and systemic barriers, contributing to lower satisfaction than their White counterparts [30]. Low satisfaction with care among SMW is linked to future healthcare behaviors, including delaying or avoiding necessary care [31]. The unique needs and experiences of subgroups—such as older SMW with lower vs. higher incomes, older SMW with poorer vs. better self-rated physical and mental health, or older SMW with more vs. less social support—remain underexplored [9]. Addressing these gaps is essential for promoting equitable, inclusive, and patient-centered care for older SMW.

### 1.4. The current study

To address these gaps, the present study explored how sociodemographic, health-related, and interpersonal factors are associated with help-seeking behavior, barriers to care, and treatment satisfaction among older SMW. Guided by syndemic theory, structural vulnerability frameworks, and Andersen’s behavioral model, we took an exploratory approach to first identify sociodemographic characteristics, self-rated physical and mental health, and interpersonal factors as correlates of specific help-seeking behaviors. We hypothesized that sociodemographic characteristics (e.g., age, income, race/ethnicity), self-rated physical and mental health, and interpersonal factors (e.g., social support, relationship status) are associated with engagement in specific help-seeking behaviors (e.g., for mental health, substance use, or memory concerns) among older SMW. Second, we examined whether general help-seeking behaviors (collapsed across specific issues) varied by older SMW’s sociodemographic characteristics, self-rated physical and mental health, and interpersonal factors, capturing predictors of broader tendencies to seek support across different concerns. We hypothesized that general help-seeking behavior varies based on older SMW’s sociodemographic characteristics, physical and mental health, and interpersonal factors. Third, we assessed whether sociodemographic characteristics, self-rated physical and mental health, and interpersonal factors were associated with help-seeking barriers. We hypothesized that older SMW with more minoritized identities, poorer self-rated health, or limited interpersonal support report greater barriers to accessing care. Fourth, we investigated associations of sociodemographic characteristics, self-rated physical and mental health, and interpersonal factors with SMW’s treatment satisfaction and satisfaction with providers. We hypothesized that older SMW with more minoritized identities, poorer self-rated health, or limited interpersonal support report lower treatment satisfaction and satisfaction with specific providers among older SMW. Findings from this study contribute needed empirical evidence to a growing but still nascent literature on the health and help-seeking experiences of aging SMW, with the goal of informing more responsive, inclusive, and effective care.

## 2. Materials and methods

### 2.1. Participants and procedures

Data from the current study are from the Chicago Health and Life Experiences of Women (CHLEW) Study [32], a community-based, 24-year, five-wave longitudinal study of SMW’s health initiated in 2000. CHLEW is the longest-running and most comprehensive study of SMW’s drinking in the U.S. or elsewhere. SMW assigned female at birth were originally recruited in the Chicago Metropolitan Area in Illinois, a midwestern state in the United States (U.S.). The CHLEW Study was designed to replicate and extend the National Study of Health and Life Experiences of Women (NSHLEW), a 20-year longitudinal study (1981, 1986, 1991, 1996, 2001) of more than 1600 women in the U.S. general population [32]. The NSHLEW was conducted in cooperation with the National Opinion Research Center [33, 34].

Wave 1 (2000–2001) of CHLEW included 447 English-speaking lesbian-identified women aged 18 and older. Wave 2 (2004–2005) reinterviewed 384 (86%) of the Wave 1 sample. Wave 3 data were collected in 2010–2012. In this wave, 353 (79%) of the original sample were retained. Modified respondent-driven sampling was used to recruit a supplemental sample of bisexual women, younger women (18–25 years), and Black and Latinx women, increasing the diversity of the sample. The final Wave 3 sample included the original (*n* = 353) and supplemental (*n* = 373; *N* = 726) samples and represents the largest and most diverse sample across the five waves of the CHLEW Study. Consistent with our prior research [35], we excluded the few women who previously identified as lesbian or bisexual, but in Wave 3 identified as mostly heterosexual, only heterosexual, or another sexual identity (*N* = 695). This approach ensures consistency in sexual identity across waves and reduces misclassification bias. Furthermore, this decision aligns with recommendations from the sexual minority health literature, which emphasizes the importance of accounting for identity fluidity while also recognizing the distinct experiences of individuals who consistently identify as lesbian or bisexual [36].

The current analyses included data from participants aged 50 years or older (*N* = 196). Data were collected via computer-assisted in-person or telephone interviews [32]. All participants provided informed consent, in accordance with the Declaration of Helsinki. The current study used data from Wave 3 of the CHLEW Study, which was approved (protocol code 2000–0714) in 13 April 2010 the University of Illinois at Chicago Institutional Review Board where the principal investigator (Hughes) held her primary appointment. The current study was approved (protocol code AAA-S3577) on 25 February 2020 by Columbia University Institutional Review Board, where Hughes currently holds her primary appointment.

### 2.2. Measures

#### 2.2.1. Help-seeking

Participants were asked about conditions for which they had sought help in the past five years, including problems related to depression, anxiety, memory, eating, or alcohol or drug use. Each item was coded 0 (no help-seeking) or 1 (any help-seeking); raw scores were combined and coded 0 (no help-seeking for mental or behavioral health problems) or 1 (help-seeking for any mental or behavioral health problem). Help-seeking variables were dichotomized as we aimed to capture the presence or absence of engagement or barriers related to any mental, cognitive, or behavioral health concern, rather than measuring gradations of frequency or intensity. While continuous measures can capture nuances in frequency or degree, dichotomization facilitated the identification of individuals at risk or in need of services, and reduced measurement noise that may have arisen from variability in the number of help-seeking instances. Additionally, given the exploratory nature of the study and the low base rates for some specific behaviors, dichotomizing variables improved statistical power and model stability. Second, inability to access needed services was defined as an affirmative response to a question asking whether, in the past five years, participants wanted or needed services for each of the above concerns but were unable to obtain such services. Each item was coded 0 (no) or 1 (yes); raw scores were combined and coded 0 (able to access needed services) or 1 (inability to access needed services). Further, collapsing across specific concerns (e.g., depression, anxiety, memory) to create a single binary indicator (0 = no help-seeking/no barrier; 1 = any help-seeking/any barrier) allowed for a more parsimonious and interpretable measure of whether participants had engaged with or faced barriers to mental and behavioral healthcare. This approach aligns with prior research that emphasizes broad patterns of healthcare access and supports the analysis of system-level engagement, consistent with the behavioral model of health service use [19]. Further, measures of help-seeking in this study were designed to replicate measures that have been validated and used in the parent study—the NSHLEW [33, 34].

#### 2.2.2. Treatment satisfaction

Treatment satisfaction was assessed by asking participants who reported having sought help for each of the above concerns to what extent their needs were met. Response options were none of my needs were met (0), only a few of my needs were met (1), most of my needs were met (2), or almost all my needs were met (3). Responses were dichotomized to reflect whether participants reported substantial unmet needs (no needs were met or only a few needs were met [0] vs. most or all needs were met [1]). Satisfaction with providers who delivered the services was assessed with the question, “Thinking about the mental health provider(s) you saw most recently, did the services… hurt you a lot (0), hurt you a little (1), make no difference (2), help you a little (3), or help you a lot (4).” Responses were dichotomized as services hurt, did not help, or made no difference (0) vs. helped at least a little (1). Treatment satisfaction variables were dichotomized, a strategy commonly used in survey-based mental health services research [37, 38] to reflect potentially clinically meaningful thresholds of perceived benefit [39–41]. Similarly to help-seeking variables, treatment satisfaction variables were designed to replicate measures that have been validated and used in the parent study, the NSHLEW [33, 34].

#### 2.2.3. Self-rated physical and mental health

Self-rated physical health in the past 12 months was assessed using continuous single-item measures. Participants were asked to rate their physical health on a scale ranging from 1 (excellent) to 6 (very poor). Similarly, self-rated mental health in the past 12 months was assessed using a question that asked participants to rate their emotional/mental health on a scale ranging from 1 (excellent) to 6 (very poor). These self-rated physical and mental health questions were modeled after global self-assessment items widely used in epidemiologic surveillance (e.g., Behavioral Risk Factor Surveillance System), which have shown strong predictive validity despite their brevity.

#### 2.2.4. Interpersonal factors

Participants reported their current relationship status as not in a committed relationship (single), in a committed relationship but not living together (not cohabiting), or in a committed relationship and living together (cohabiting). We combined SMW in a committed relationship but not living together (not cohabiting) and SMW in a committed relationship and living together (cohabiting) as we found no differences between the two groups (data available upon request). We then dichotomized as single or not currently in a relationship (0) vs. currently in a committed relationship regardless of cohabitation status (1). Social support was assessed using the 12-item Multidimensional Scale of Perceived Social Support (MSPSS) [42]; we used total social support scores (Cronbach’s *α* = 0.93). Prior research has shown MSPSS to have strong internal consistency for the overall scale among SMW (Cronbach’s *α* = 0.88) [38].

#### 2.2.5. Sociodemographic characteristics

We measured age (in years) continuously. Participants reported their sexual identity as only lesbian/gay, mostly lesbian/gay, bisexual, mostly heterosexual, only heterosexual/straight, or other. Consistent with prior research [38], and because responses to key questions differed little between SMW who identified as mostly lesbian/gay versus only lesbian/gay, we created a dummy variable to represent those who identified as only lesbian/gay or mostly lesbian/gay (0; lesbian) or bisexual (1). Race/ethnicity was assessed using responses to two questions assessing Hispanic or Latinx origin or heritage and race. Those who identified as both Latinx and any other group were categorized as Latinx. Those who identified with more than one race/ethnicity or as a race/ethnicity other than White, Black, or Latinx (n = 5) were excluded from the current analyses given small cell sizes due to the risk of unreliable parameter estimates and inflated standard errors. Response options were coded as non-Hispanic White (0), non-Hispanic Black (1), and Latinx (2). SMW were asked whether their current household income was enough to meet their basic needs. Response options were not enough to meet basic needs (1), enough to meet basic needs (2), or more than enough to meet basic needs (3). Response options were coded as 0 (sufficient income to meet basic needs) vs. 1 (insufficient income to meet basic needs).

### 2.3. Statistical analysis

Descriptive statistics (i.e., means, standard deviations, and proportions) were used to summarize the sample’s demographics. Bivariate analyses, adjusted for sociodemographic characteristics, were conducted to examine associations among help-seeking behaviors for mental and behavioral health concerns (i.e., problems related to depression, anxiety, memory, eating, alcohol use, and other drug use), self-rated physical and mental health, relationship status, and social support. We used multivariate logistic regression models to examine the associations between sociodemographic characteristics and help-seeking for these issues.

We then used separate multivariable logistic regression models to assess associations between help-seeking and treatment satisfaction variables (i.e., inability to access needed services, treatment satisfaction, satisfaction with providers) and age, sexual identity, race/ethnicity, and income (Model 1). In Model 2, we examined self-rated physical health and self-rated mental health as predictors of help-seeking and treatment satisfaction. Model 3 regressed help-seeking and treatment satisfaction on interpersonal factors. Across all adjusted models, we controlled for sociodemographic characteristics.

Sample sizes in each model varied based on missing data. Missing data for explanatory variables and covariates ranged from 0 for age to 5 (2.6%) for social support and were handled using pairwise deletion. Only SMW who reported having sought help for behavioral or mental health concerns (i.e., depression, anxiety, memory, eating, alcohol use, and drug use) were included in the treatment satisfaction models. That is, 37.2% of the total sample were included in the treatment satisfaction models, and 38.3% of the total sample were included in the provider satisfaction models.

We present exponentiated coefficients (odds ratios [ORs], adjusted odds ratios [aORs]), 95% confidence intervals (CIs), and *p*-values. Analyses were conducted in SPSS version 29.

## 3. Results

As shown in Table 1, participants (*N* = 196) were, on average, 58.15 years of age (*SD* = 6.61; range = 50 to 82). The majority identified as lesbian (88.3%). A third of SMW identified as Black (33.7%); fewer (15.3%) identified as Latinx. More than a third (34.7%) of SMW reported that their annual household income was not enough to meet basic needs. Most participants (60.2%) were in a relationship. Less than half of participants (43.4%) sought help for mental or behavioral health problems in the past five years; most (35.2%) reported seeking help for depression symptoms, followed by anxiety symptoms (25.0%). Fourteen (7.1%) participants reported being unable to access needed services. Of the 85 participants who reported seeking help, only 31.6% reported that most or all their needs were met. More than a third (*n* = 71; 36.2%) reported that their providers helped at least a little.

### 3.1. Help-seeking behaviors, self-rated health, and interpersonal factors

As shown in Table 2, SMW who sought treatment for depression (vs. those who did not) were more likely to seek help for anxiety, memory-related problems, and eating-related problems. SMW who sought treatment for anxiety (vs. those who did not) were more likely to seek treatment for memory problems and eating problems. Those who sought help for memory problems (vs. those who did not) were more likely to seek help for eating problems. SMW who sought treatment for depression (vs. those who did not) were not more likely to seek treatment for alcohol problems or drug problems. SMW who sought treatment for anxiety (vs. those who did not) were not more likely to seek treatment for alcohol problems or drug problems. SMW who sought treatment for memory problems (vs. those who did not) were not more likely to seek treatment for alcohol problems or drug problems. SMW who sought treatment for eating problems (vs. those who did not) were not more likely to seek treatment for alcohol problems. The model did not converge when examining the association between help-seeking for eating problems and help-seeking for drug programs, likely due to small cell sizes. SMW who sought treatment for alcohol problems (vs. those who did not) were not more likely to seek treatment for drug problems.

SMW who reported poorer physical health were more likely to seek treatment for depression, anxiety, and memory problems. SMW who reported poorer mental health were more likely to seek treatment for depression, anxiety, and memory problems. SMW in relationships reported better social support than those not in a relationship, and self-rated poorer mental health was associated with poorer physical health and less social support.

### 3.2. Sociodemographic characteristics associated with help-seeking for specific issues

SMW who reported insufficient (vs. sufficient) income were more likely to report seeking help for depression, anxiety, and memory problems in the past five years (see Table 3). Help-seeking for depression, anxiety, or memory problems were not associated with participants’ age, sexual identity, or race/ethnicity. Sociodemographic characteristics were not associated with seeking help for eating problems. Bisexual women and Black SMW were more likely than lesbian women and White SMW, respectively, to seek help for alcohol problems, but seeking help for alcohol problems did not vary by age or income. SMW who were older were less likely to seek help for drug problems than their younger counterparts. Those who reported having insufficient income were more likely to seek help for drug problems than those with sufficient income. Help-seeking for drug problems did not vary by sexual identity or race/ethnicity.

### 3.3. Predictors of help-seeking in general and treatment satisfaction

SMW with insufficient household income (vs. sufficient income) to meet basic needs were more likely to seek treatment for problems related to depression, anxiety, memory, eating, alcohol use, and drug use (see Table 4). SMW who reported poorer physical health and poorer mental health were more likely to seek treatment for mental and behavioral health problems; this finding held after adjusting for sociodemographic characteristics. We found no differences in help-seeking based on age, sexual identity, race/ethnicity, or interpersonal factors.

Adjusting for age, sexual identity, and income, Black SMW and Latinx SMW were less likely than White SMW to report inability to access needed services for mental and behavioral health problems. SMW who reported poorer mental health were more likely to report inability to access needed services; this finding did not hold when not adjusting for sociodemographic characteristics. We found no differences in inability to access needed services based on age, sexual identity, income, self-rated physical health, or interpersonal factors.

Participants who reported poorer mental health were less likely to report satisfaction with treatment; this model adjusted for sociodemographic characteristics and poor physical health. Compared to participants with less social support, those with more social support had higher odds of reporting satisfaction with treatment; this finding did not hold when adjusting for sociodemographic characteristics and relationship status. Treatment satisfaction did not vary based on age, sexual identity, race/ethnicity, income, physical health, or relationship status.

Compared to participants with less social support, those with more social support were more likely to report satisfaction with providers; this finding held over and above the effects of sociodemographic characteristics and relationship status. We found no differences in satisfaction with providers based on age, sexual identity, race/ethnicity, income, self-rated physical health or mental health, or relationship status.

## 4. Discussion

Our findings highlight complex factors influencing help-seeking behaviors, barriers to care, and treatment satisfaction among older SMW, informed by syndemic [10, 11, 43] and structural vulnerability frameworks [17, 44] and Andersen’s behavioral health care utilization model [19]. Specifically, older SMW with insufficient income and self-reported poorer physical and mental health were more likely to seek help for problems related to depression, anxiety, memory, eating, and alcohol and other drug use than those with sufficient income and self-reported better physical and mental health. White SMW and those with self-reported poorer mental health reported more difficulties accessing services compared to Black older SMW and those who reported better mental health. Older SMW who self-reported better mental health and greater social support reported higher treatment satisfaction. Social support also related to greater satisfaction with treatment providers.

Building on research documenting health disparities linked to sexual identity among older women [3, 4, 43] our study examined how physical health, problems related to mood (i.e., depression and anxiety), eating-related problems, memory issues, and substance use may be interconnected in this population. We found that older SMW who sought treatment for problems related to depression or anxiety were more likely to also seek help for memory problems and eating problems, supporting prior findings that these issues are synergistic and frequently co-occur among older SMW [11, 43, 45]. These patterns underscore the need for more holistic and affirming care models that attend to the full range of interconnected mental health and neurocognitive concerns facing older SMW.

However, not all types of help-seeking were associated. Specifically, SMW who sought treatment for problems related to depression, anxiety, memory, or eating were not more likely to seek treatment for problems related to alcohol or drug use. These findings suggest that mood-, cognitive-, and eating-related concerns may be more tightly linked in ways that prompt older SMW to seek care across those domains, perhaps due to overlapping symptoms (e.g., negative affect, difficulty concentrating), shared underlying causes (e.g., stress exposure, social isolation), or more integrated pathways to care (e.g., primary care screening for both depression and cognitive issues) [46–48]. Indeed, older SMW were less likely to seek care for substance use alongside these issues, even though they tend to co-occur frequently in this population [43].

Older SMW may experience or perceive stigma from providers, shame, fragmented care systems, or other structural barriers that may make it more difficult to access mental health, neurocognitive, and substance use treatment in tandem [46, 49]. The lack of significant associations does not necessarily mean these issues do not co-occur; rather, that they may not co-occur to a degree that leads to integrated help-seeking—or they may be addressed through separate, uncoordinated pathways [46, 50]. Further research is needed to clarify whether these patterns reflect differences in symptom presentation, shared etiology, care access, or perceptions of need for treatment, particularly for co-occurring mental health, cognitive functioning, and substance use among older SMW.

Patterns of help-seeking varied based on intersecting social and structural factors, including income, age, race, and sexual identity. Income insufficiency was associated with greater help-seeking for depression, anxiety, memory problems, and drug use. Bisexual and Black older SMW were more likely than their lesbian and White peers to seek help for alcohol-related issues. SMW who were younger were more likely to seek help for drug problems than those who were older, reflecting the impact of structural vulnerabilities and intersecting social determinants of health among older SMW [17, 44]. Income insufficiency can elevate health risks and subsequent help-seeking behaviors by increasing stress, limiting access to preventative care, and necessitating greater reliance on health services [51]. Bisexual and Black older SMW face intersecting stigma-related stressors—such as biphobia, racism, sexism, and ageism—that contribute to alcohol-related challenges and may motivate help-seeking for these issues [43, 52].

SMW with poorer physical and mental health were more likely to seek treatment for depression, anxiety, and memory problems, even after adjusting for sociodemographic characteristics. These findings are consistent with Andersen’s model [19], which links perceived need, based on the severity of health conditions, to help-seeking behavior. This pattern also aligns with prior research showing that older adults with chronic health conditions are more likely to seek care [20, 21]. Among older SMW, poorer mental health was associated with poorer physical health and reduced social support. Inadequate support may limit access to emotional and tangible resources that could buffer identity- and age-related stress, conferring risk for compounding health issues and ultimately greater help-seeking [53].

Building on prior work [9], we found that help-seeking barriers varied among older SMW, as Black and Latinx SMW were less likely to report such barriers. While this finding may appear unexpected given structural racism and well-documented health inequities, it aligns with prior studies suggesting that SMW of color may draw on psychological resources (e.g., identity affirmation), navigational and social capital (e.g., participation in social activities), and spirituality to overcome barriers to care [18]. It is also possible that differences in expectations shape perceptions of access—for example, White SMW may anticipate smoother pathways to care due to structural privilege, making them more likely to perceive and report barriers when care falls short. In contrast, SMW of color may have different reference points based on lifetime experiences with systemic marginalization, which could influence how they assess and report barriers [25, 44].

SMW with poorer mental health were more likely to report barriers to accessing needed care, potentially reflecting the need for more intensive or specialized care (e.g., trauma-informed therapy, culturally competent providers), which is often more difficult to access [46, 54–57]. Poorer mental health can also heighten sensitivity to perceived or actual stigma related to mental health disorders or minoritized identities, contributing to care-access barriers [58]. Notably, after accounting for sociodemographic variables, the impact of self-rated mental health on barriers to care was less pronounced. These findings, aligned with vulnerability frameworks [44, 55], suggest that social and structural factors, such as income insufficiency and potentially access to health insurance, may play a larger role in older SMW’s ability to access mental and behavioral health care.

Self-rated poorer mental health was associated with lower treatment satisfaction among older SMW, aligned with broader research among older adults that demonstrates a connection between poor mental health and dissatisfaction with provider–patient interactions and overall care [59]. Older SMW with mental health concerns may encounter identity- and mental health-related stigma, symptom minimization, or negative provider perceptions [27, 58]. These negative interactions may reduce older SMW’s involvement in treatment decision-making and lower treatment satisfaction, likely due to decreased trust in providers [59]. In addition, older SMW with greater mental health symptom burden may enter treatment with heightened expectations for relief [60]. Treatment satisfaction may decline when treatment expectations and experiences are misaligned [61]. Future research should examine how treatment preferences, expectations, and experiences influence satisfaction among older SMW with poorer mental health.

Social support, though positively associated with treatment satisfaction, had a weaker effect after adjusting for sociodemographic characteristics and relationship status. According to Andersen’s model [19], although social support is key for accessing care, treatment satisfaction may be more influenced by needs, expectations, and care quality. Thus, the unique contribution of social support to treatment satisfaction (as opposed to access) may be weaker but still present after accounting for sociodemographic factors (e.g., income, education) and relationship status. However, social support remained a consistent predictor of satisfaction with providers. The availability of support may help older SMW manage health conditions, improve treatment adherence, and enhance well-being and coping self-efficacy, ultimately enhancing care and provider satisfaction [53]. Moreover, social support may strengthen the therapeutic alliance, a key factor in provider satisfaction, more so than objective effectiveness of care (e.g., symptom relief, goal achievement), which may be more closely linked to treatment satisfaction [62].

### 4.1. Practice and policy implications

Our findings underscore the need to improve access to effective, integrated, and affirming care for older SMW, as unmet health needs contribute to diminished quality of life, and increased morbidity [15, 63]. This study highlights the need to comprehensively assess and provide coordinated treatment of mental, behavioral, cognitive, and physical health concerns in this population. Structural stressors, including income insufficiency, sexism, homophobia/biphobia, racism, and ageism, may exacerbate health conditions by elevating stress, limiting preventative care utilization, and increasing reliance on health services to manage chronic conditions [64].

Clinicians should be equipped to recognize the compounded impact of economic disparities and discrimination and incorporate culturally and structurally competent practices into routine care, as research has demonstrated persistent gaps in provider training and preparedness to deliver culturally responsive services to sexual and gender minority populations [65]. This may include regular training in intersectional, syndemic-informed, and affirming approaches. Services tailored to the unique needs of bisexual and Black SMW and those with insufficient income are especially needed to reduce access barriers and improve treatment outcomes. Enhancing provider communication and patient advocacy—alongside efforts to strengthen social support networks through peer navigation and community-based resources—may further promote treatment and provider satisfaction among SMW [66].

Policymakers should prioritize increasing access to affordable and comprehensive health coverage, particularly for those with income insufficiency, as financial vulnerability emerged as a consistent correlate of both help-seeking and unmet need. Macro-level policies should reduce structural inequities by funding programs that reduce barriers to care, incorporating input from older SMW to guide resource allocation. To strengthen health systems, policymakers could create incentives for insurance companies and providers to implement comprehensive and integrated care models [67]. These incentives could support routine and coordinated screening for mental health, cognitive concerns, physical health issues, and substance use, alongside culturally tailored prevention efforts [68].

### 4.2. Limitations and directions for future research

Several limitations should be considered. Although the original sample, recruited in the greater Chicago Metropolitan Area, was more geographically dispersed by Wave 3, most participants remained in the area, limiting generalizability. Although the data were collected between 2010 and 2012, the study addresses a significant gap in research on help-seeking and satisfaction among SMW aged 50 and older, a population that remains underrepresented in mental and behavioral health services research. However, findings may not fully capture shifts in policy, social climate, or healthcare access over the past decade. Additionally, the data were collected in the United States, which limits the generalizability of findings to other countries with different healthcare systems, sociopolitical environments, and cultural norms regarding sexual minority individuals and aging.

In addition, 88.3% of the sample identified as lesbian, which is common among older SMW [69], but may not reflect the experiences of older SMW with other sexual identities, such as pansexual or queer. Collapsing responses into binary categories (lesbian vs. bisexual) may obscure within-group differences, such as the experiences of mostly lesbian/gay women. The cross-sectional design limits the ability to address temporal sequencing of study variables or changes in help-seeking behaviors, barriers, and satisfaction over time. Future research would benefit from applying structural equation modeling or related techniques to examine potential pathways and mediating mechanisms underlying these relationships.

Retrospective self-report measures and single-item questions may reduce sensitivity, and important factors like duration of service use and provider characteristics were not assessed. Although help-seeking, treatment satisfaction, and self-rated mental and physical health questions were not pilot-tested formally in advance of the current study, they were reviewed by our multidisciplinary team to maximize clarity, cultural relevance, and acceptability. Item brevity was a key consideration given the longitudinal nature of CHLEW Study and the need to minimize participant burden across multiple waves of data collection. Yet, future studies would benefit from more formal validation of help-seeking, treatment satisfaction, and self-rated mental and physical health variables or the incorporation of validated scales where feasible. Although dichotomizing variables related to help-seeking, access to care, and treatment satisfaction improved interpretability and addressed analytic constraints (e.g., small sample, skewed distribution), this approach may have obscured meaningful variability in participants’ experiences; future research with larger samples should explore these constructs using continuous or ordinal measures to capture greater nuance. Moreover, help-seeking for other mental health concerns (e.g., problems related to trauma or psychosis) was not captured in the current study but should be assessed in future research.

More research using intersectional analyses to explore help-seeking behaviors and barriers among older SMW with multiple minoritized identities is needed. Including older SMW with diverse sexual and gender identities, and other systemically marginalized racial and ethnic identities, would provide a more nuanced understanding of how experiences related to these minoritized identities influence help-seeking and satisfaction. Examining social and structural determinants of health, such as trauma and structural stigma, respectively, could also provide insights into healthcare utilization, help-seeking barriers, and treatment satisfaction in this population. Longitudinal research is needed to assess the temporal relationship between mental health and treatment satisfaction, as lower satisfaction might exacerbate symptoms. Research is needed that considers factors like patient–provider communication, identity concealment and level of outness, shared treatment decision-making, and self-efficacy as potential influences on older SMW’s care satisfaction [28]. Future studies should incorporate validated measures of masculinity and femininity to assess how gender expression, gendered experiences, and conformity to gender norms may intersect with other sociodemographic factors to influence service use and treatment satisfaction, especially among older SMW for whom gender expression and identity may diverge from traditional binaries. Further, future studies should explore how caregiving networks and family structure, including the presence or absence of adult children, may shape older SMW’s engagement with healthcare services and treatment satisfaction, given the known influence of adult children on service use [48].

## 5. Conclusions

The present study highlights the complex interplay of structural and health-related factors, including income insufficiency, stigma-related experiences due to identifying as bisexual, and poor self-rated physical and mental health, in shaping older SMW’s need for comprehensive health services. Findings demonstrate significant disparities in care barriers and satisfaction, driven by intersecting challenges, such as poorer mental health and limited social support. Addressing these issues requires integrated, culturally competent, and preventative care models that account for older SMW’s syndemic conditions and enhance treatment access. Efforts to reduce help-seeking barriers and improve treatment satisfaction should focus on patient-centered approaches, leverage existing strengths (e.g., social support), promote advocacy, improve provider training, and implement policies addressing systemic inequities for older SMW. Future research should prioritize longitudinal and mixed-methods studies with diverse aging SMW to capture evolving comorbid health needs and help-seeking experiences. Finally, as telehealth becomes a more common mode of care delivery, future research should examine whether virtual platforms reduce stigma or increase satisfaction and service engagement among older SMW, particularly those with complex health needs or limited access to affirming care.

## Figures and Tables

**Table 1 •:** Sample characteristics in sexual minority women 50 years of age or older (Chicago Health and Life Experiences of Women; *N* = 196).

	*n* (%)
**Sociodemographic Characteristics**	
Age, *M* [SD] (Range: 50–82)	58.15 [6.61]
**Sexual Identity**	
Bisexual	23 (11.7)
Lesbian	173 (88.3)
**Race/Ethnicity**	
Black	66 (33.7)
Latinx	30 (15.3)
White	95 (48.5)
**Income**	
Annual income ≥ USD 40,000	108 (55.1)
Annual income ≥ USD 15,000 and <USD 40,000	38 (19.4)
Annual income ≥ USD 5000 and <USD 15,000	31 (15.8)
Annual income < USD 5000	13 (6.6)
**Income sufficiency**	
Income sufficient to meet basic needs	124 (63.3)
Income insufficient to meet basic needs	68 (34.7)
**Help-Seeking**	
Help-seeking for alcohol problems	9 (4.6)
Help-seeking for anxiety symptoms	49 (25.0)
Help-seeking for depression symptoms	69 (35.2)
Help-seeking for drug problems	7 (3.6)
Help-seeking for eating problems	12 (6.1)
Help-seeking for memory problems	20 (10.2)
**Self-Rated Physical and Mental Health**	
Self-rated physical health, *M* [SD], (Range: 1–6)	2.86 [1.26]
Self-rated mental health, *M* [SD], (Range: 1–6)	2.78 [1.27]
**Interpersonal Factors**	
**Relationship status**	
In a relationship	118 (60.2)
Not in a relationship	78 (39.8)
Social support, *M* [SD] (Range: 18–84)	65.84 [14.31]

**Notes:** Percentages may not equal 100 due to missing data. Abbreviations: *n*, size of a subgroup; *M*, mean; SD, standard deviation.

**Table 2 •:** Bivariate associations among help-seeking behaviors, self-rated physical health, self-rated mental health, and social support.

	Help-seeking for depression problems aOR (95% CI)	Help-seeking for anxiety problems aOR (95% CI)	Help-seeking for memory problems aOR (95% CI)	Help-seeking for eating problems aOR (95% CI)	Help-seeking for alcohol problems aOR (95% CI)	Help-seeking for drug problems aOR (95% CI)	Self-rated physical health aOR (95% CI)	Self-rated mental health aOR (95% CI)	Relationship status aOR (95% CI)	Social support aOR (95% CI)
1. Help-seeking for depression problems	—	**17.19** *** **(7.23, 40.90)**	**12.39** *** **(3.37, 45.49)**	**5.74** * **(1.38, 23.89)**	1.29 (0.29, 5.64)	9.70 (0.97, 97.24)	**1.53** ** **(1.16, 2.03)**	**2.06** *** **(1.52, 2.79)**	0.65 (0.34, 1.26)	0.99 (0.97, 1.01)
2. Help-seeking for anxiety problems		—	**11.75** *** **(3.88, 35.60)**	**5.80** *** **(1.57, 21.42)**	1.45 (0.32, 6.55)	1.29 (0.22, 7.60)	**1.56** ** **(1.16, 2.11)**	**1.85** *** **(1.37, 2.51)**	1.07 (0.53, 2.19)	0.99 (0.96, 1.01)
3. Help-seeking for memory problems			—	**18.19** *** **(4.10, 80.63)**	2.15 (0.37, 12.65)	5.34 (0.84, 33.84)	**2.39** *** **(1.47, 3.91)**	**1.91** *** **(1.28, 2.86)**	2.89 (0.92, 9.13)	0.99 (0.95, 1.02)
4. Help-seeking for eating problems				—	2.12 (0.23, 19.96)	—	1.27 (0.76, 2.11)	1.44 (0.87, 2.38)	3.68 (0.74, 18.29)	1.01 (0.96, 1.05)
5. Help-seeking for alcohol problems					—	2.53 (0.13, 48.59)	1.87 (0.96, 3.63)	1.03 (0.57, 1.87)	1.60 (0.34, 7.55)	1.01 (0.96, 1.07)
6. Help-seeking for drug problems						—	1.88 (0.87, 4.08)	1.11 (0.55, 2.21)	1.27 (0.19, 8.49)	1.01 (0.95, 1.07)
7. Self-rated physical health							—	**0.39** ***	1.08 (0.88, 1.32)	−0.12
8. Self-rated mental health								—	0.84 (0.65, 1.08)	**−0.20** **
9. Relationship status									—	**1.06** *** **(1.03, 1.09)**
10. Social support										—

**Notes:** ORs with 95% confidence intervals adjusted for age, sexual identity, race/ethnicity, and income. Boldface type indicates a significant adjusted odds ratio. Associations between specific reasons for help-seeking, and between help-seeking behaviors and relationship status were tested using bivariate logistic regressions. Associations among self-rated physical health, self-rated mental health, and social support were tested using bivariate correlations.

* *p* < 0.05,

** *p* < 0.01,

*** *p* < 0.001. Abbreviations: aOR, adjusted odds ratio; OR, odds ratio; CI, confidence interval.

**Table 3 •:** Multivariable logistic regression analyses assessing sociodemographic characteristics and help-seeking behaviors for various mental and behavioral health issues.

	Help-seeking for depression problems aOR (95% CI)	Help-seeking for anxiety problems aOR (95% CI)	Help-seeking for memory problems aOR (95% CI)	Help-seeking for eating problems aOR (95% CI)	Help-seeking for alcohol problems aOR (95% CI)	Help-seeking for drug problems aOR (95% CI)
**Model 1**						
**Sociodemographic Characteristics**						
Age	0.96 (0.92, 1.01)	0.98 (0.93, 1.03)	0.99 (0.93, 1.07)	0.99 (0.90, 1.08)	0.95 (0.84, 1.07)	**0.75** * **(0.56, 0.99)**
Sexual identity						
Lesbian	*ref*	*ref*	*ref*	*ref*	*ref*	*ref*
Bisexual	0.98 (0.39, 2.44)	0.81 (0.29, 2.32)	1.38 (0.37, 5.12)	0.67 (0.08, 5.44)	**4.53** * **(1.04, 19.77)**	3.20 (0.58, 17.54)
Race/Ethnicity						
White	*ref*	*ref*	*ref*	*ref*	*ref*	*ref*
Black	0.96 (0.49, 1.89)	0.47 (0.21, 1.07)	1.51 (0.57, 4.03)	1.47 (0.35, 6.09)	**6.64** * **(1.33, 33.19)**	7.71 (0.88, 67.56)
Latinx	1.58 (0.68, 3.66)	1.54 (0.64, 3.66)	0.68 (0.14, 3.35)	2.53 (0.53, 11.99)	—	3.24 (0.19, 53.46)
Income						
Income sufficient to meet basic needs	*ref*	*ref*	*ref*	*ref*	*ref*	*ref*
Income insufficient to meet basic needs	**3.10** *** **(1.67, 5.77)**	**2.16** * **(1.11, 4.19)**	**3.11** * **(1.20, 8.03)**	1.33 (0.40, 4.35)	1.54 (0.39, 5.97)	**11.90** * **(1.40, 101.06)**

**Notes:** Boldface type indicates a significant adjusted odds ratio.

* *p* < 0.05,

*** *p* < 0.001. Abbreviations: aOR, adjusted odds ratio; CI, confidence interval; ref, reference group.

**Table 4 •:** Multivariable logistic regression analyses assessing sociodemographic characteristics, self-rated physical and mental health, interpersonal factors, help-seeking, and treatment satisfaction in separate models.

	Help-seeking for mental and behavioral health problems ^a^		Inability to access needed services ^b^		Treatment satisfaction ^c^		Satisfaction with providers ^d^	
	OR (95% CI)	aOR (95% CI)	OR (95% CI)	aOR (95% CI)	OR (95% CI)	aOR (95% CI)	OR (95% CI)	aOR (95% CI)
**Model 1**								
**Sociodemographic Characteristics**								
Age	0.96 (0.91, 1.01)		0.89 (0.76, 1.05)		0.98 (0.88, 1.10)		0.89 (0.77, 1.03)	
Sexual identity								
Lesbian	*ref*		*ref*		*ref*		*ref*	
Bisexual	1.23 (0.45, 3.34)		0.32 (0.03, 2.88)		0.30 (0.05, 1.95)		—	
Race/Ethnicity								
White	*ref*		*ref*		*ref*		*ref*	
Black	0.59 (0.27, 1.27)		**0.10** * **(0.01, 0.70)**		0.92 (0.17, 5.03)		0.38 (0.05, 2.92)	
Latinx	0.72 (0.27, 1.90)		**0.05** * **(0.01, 0.62)**		0.36 (0.07, 1.88)		0.15 (0.02, 1.29)	
Income								
Income sufficient to meet basic needs	*ref*		*ref*		*ref*		*ref*	
Income insufficient to meet basic needs	**3.15** *** **(1.59, 6.24)**		0.50 (0.12, 2.11)		1.05 (0.26, 4.23)		0.57 (0.11, 2.86)	
**Model 2**								
**Self-Rated Physical and Mental Health**								
Self-rated physical health	**1.67** *** **(1.26, 2.21)**	**1.71** *** **(1.24, 2.35)**	1.13 (0.65, 1.95)	1.72 (0.81, 3.64)	0.63 (0.39, 1.01)	0.67 (0.35, 1.26)	0.55 (0.30, 1.04)	0.81 (0.36, 1.81)
Self-rated mental health	1.70 *** **(1.28, 2.25)**	1.61 ** (1.18, 2.18)	**2.08** * **(1.15, 3.76)**	1.66 (0.79, 3.49)	0.67 (0.41, 1.10)	**0.53** * **(0.29, 0.96)**	0.70 (0.39, 1.27)	0.49 (0.23, 1.04)
**Model 3**								
**Interpersonal Factors**								
Relationship status								
Not in a relationship	*ref*		*ref*		*ref*		*ref*	
In a relationship	0.95 (0.50, 1.79)	1.09 (0.55, 2.17)	1.28 (0.35, 4.70)	1.82 (0.36, 9.13)	1.76 (0.54, 5.79)	1.48 (0.42, 5.23)	1.09 (0.23, 5.14)	1.29 (0.22, 7.53)
Social support	0.99 (0.97, 1.01)	0.99 (0.97, 1.02)	0.99 (0.96, 1.04)	0.96 (0.91, 1.01)	**1.06** * **(1.01, 1.10)**	1.04 (0.99, 1.09)	**1.08** ** **(1.02, 1.14)**	**1.09** * **(1.02, 1.18)**

**Notes:** Adjusted models accounted for age, sexual identity, race/ethnicity, and income. Boldface type indicates a significant odds ratio or adjusted odds ratio.

a Help-seeking for mental or behavioral health problems (1 = participants who endorsed seeking help for problems related to depression, anxiety, memory, drug use, alcohol use, or eating; 0 = participants who did not endorse seeking help for problems related to depression, anxiety, memory, drug use, alcohol use, or eating).

b Inability to access needed services (1 = participants who reported having problems related to depression, anxiety, memory, drug use, alcohol use, or eating for which they wanted or needed services that they were unable to obtain; 0 = participants who reported not having problems related to depression, anxiety, memory, drug use, alcohol use, or eating for which they wanted or needed services that they were unable to obtain).

c Treatment satisfaction (1 = participants who reported that at least most or all of their needs were met when they sought treatment for problems related to depression, anxiety, memory, drug use, alcohol use, or eating; 0 = participants who reported that none or only a few of their needs were met when they sought treatment for problems related to depression, anxiety, memory, drug use, alcohol use, or eating).

d Satisfaction with mental health providers (1 = participants who reported that their provider(s) helped them at least a little; 0 = participants who reported that their provider(s) hurt or did not help them).

* *p* < 0.05,

** *p* < 0.01,

*** *p* < 0.001. Abbreviations: aOR, adjusted odds ratio; OR, odds ratio; CI, confidence interval; ref, reference group.

## Data Availability

All data supporting the findings of this publication are available within this article.
